# Deep learning analysis of capillary refill dynamics in ischemic colitis: differentiating reversible vs. gangrenous mucosa

**DOI:** 10.3389/fgstr.2026.1782255

**Published:** 2026-07-06

**Authors:** Zarqa Yasin, Hamza Sajid, Noor ul Ain Saleem, Ursula Abu Nahla

**Affiliations:** 1 Henry Ford Jackson Hospital, Michigan, MD, United States; 2 Allama Iqbal Medical College, Lahore, Pakistan; 3 Fatima Memorial Hospital (FMH) College of Medicine and Dentistry, Lahore, Pakistan; 4 Faculty of Medicine, Hebron University, Hebron, Palestine

**Keywords:** artificial intelligence, capillary refill, colonoscopy, deep learning, ischemic colitis, perfusion metrics

## Abstract

Ischemic colitis remains a diagnostic challenge, as endoscopic grading of mucosal ischemia is subjective and insensitive for transmural infarction. While the majority of non-gangrenous cases are resolved conservatively, gangrenous mucosa necessitates immediate resection. The current reliance on visual signals, such as color and bleeding, results in misclassification and variability. We propose a novel deep learning approach to quantify micro-capillary refill dynamics from colonoscopy video. The technology produces objective perfusion measures and real-time viability classification by examining frame-by-frame color recovery following temporary mucosal blanching. This approach makes use of recent developments in AI-assisted colonoscopy, annotated datasets, and endoscopic hardware. By incorporating capillary refill analysis, operational decision-making might be improved, interobserver variability could be decreased, and unnecessary colectomy and delayed gangrene treatment could be avoided. Pilot studies are warranted to validate this concept and establish its role in guiding therapy for ischemic colitis.

## Introduction

Ischemic colitis (IC) is often graded visually during colonoscopy, but endoscopic findings lack specificity, except for frank gangrene.¹ Most non-gangrenous IC cases resolve with conservative care, whereas true mucosal infarction demands urgent resection. In practice, differentiating reversible mucosal ischemia from impending necrosis based on visual assessment is highly subjective. (1) For example, a meta-analysis after aortic surgery found endoscopy is poor at detecting transmural infarction (sensitivity ~52%). (2) This diagnostic ambiguity can lead to delayed treatment of gangrene or unnecessary colectomy of salvageable tissue.

The key challenge in IC is the subjective interpretation of mucosal color and bleeding at colonoscopy. (1) We propose a real-time computer vision tool to quantify micro-capillary refill time on colonoscopy video, providing an objective perfusion metric. During colonoscopy, brief pressure on the mucosa (via the scope tip) will transiently blanch the tissue; the time required for color restoration (“refill time”) correlates with microvascular flow. By analyzing each video frame after release, a deep learning model could extract pixel-wise color recovery curves.

The proposed pipeline involves acquiring high-resolution color video, segmenting the pressed mucosal region using machine-learning methods or optical markers, and applying color calibration to correct for lighting variability. Following automated detection of pressure release, frame-by-frame changes in red or infrared intensity are analyzed to extract quantitative refill metrics such as time to 50% color return or the area under the intensity–time curve. These spatiotemporal features are then processed by a deep-learning model trained on labeled examples of viable and gangrenous tissue to generate a binary viability classification or continuous perfusion score. The results are displayed in real time by overlaying refill metrics on the endoscopic view, with non-viable regions highlighted and alerts generated to prompt surgical consultation when indicated.

**Figure 1:**
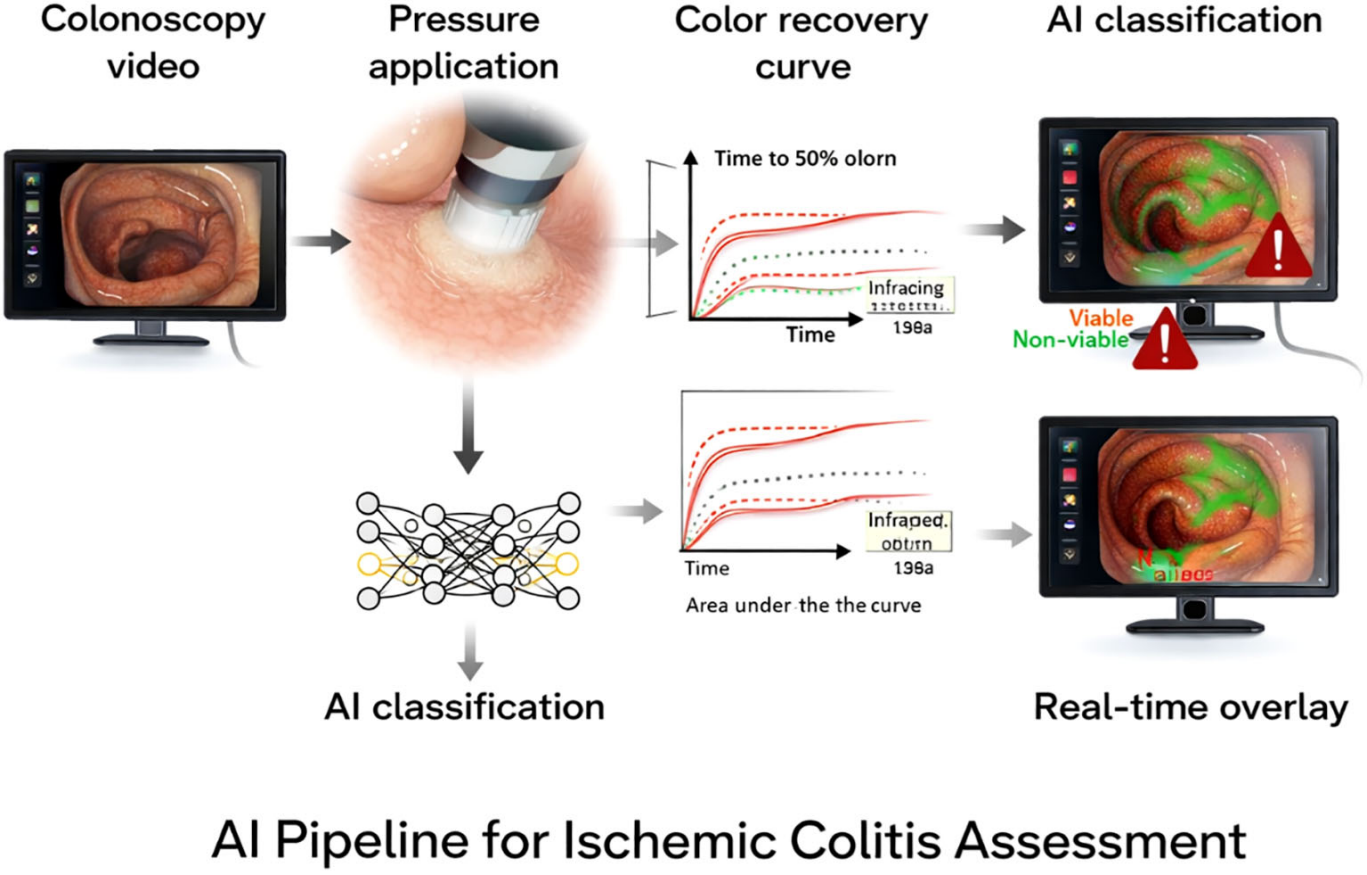
AI pipeline for ischemic colitis assessment.

Recent advances make this feasible. AI systems in colonoscopy already operate in real time, as shown in **Figure 1**. (3) Annotated video datasets (like those used for polyp detectors) can be repurposed with expert labels for ischemia. Intra-procedural tools such as multispectral single-snapshot imaging (SSOP) have demonstrated accurate tissue oxygenation maps of bowel in pigs, (4) proving that non-invasive imaging can gauge perfusion. A video-based approach is far simpler as it requires only a standard endoscope camera and software, not extra hardware.

By applying computer vision and deep learning to capillary refill, the system provides standardized perfusion metrics for each colonic segment. This can greatly reduce interobserver variability. (5) In practice, an endoscopist would see an on-screen timer or color-coded map of refill times as the scope withdraws. A delayed refill reading would trigger an alert. Such feedback augments clinical judgment: unlike arbitrary color hues or bleeding, the tool quantifies perfusion. Ultimately, this should sharpen the decision of “operate vs observe, “ avoiding needless colectomy when blood flow is intact.

In conclusion, integrating AI into colonoscopy to measure micro-capillary refill is a novel concept. It leverages the sensitivity of video analysis to deliver a robust perfusion signal, building on proof-of-concept in optical imaging and the success of AI in endoscopy. (3, 4, 6) Further clinical research is warranted to pilot this approach in ischemic colitis to guide therapy and objectively improve patient outcomes.

## Data Availability

The original contributions presented in the study are included in the article/supplementary material. Further inquiries can be directed to the corresponding author.
